# Histopathological pattern of primary bone tumours at the Black Lion Specialized Hospital, Addis Ababa, Ethiopia: a retrospective cross-sectional, 2015-2019

**DOI:** 10.11604/pamj.2022.41.62.27905

**Published:** 2022-01-21

**Authors:** Tufa Gemechu Weyessa, Endeshaw Asaye Kindie, Ermias Teklehaimanot Yefter

**Affiliations:** 1 Department of Pathology, Addis Ababa University College of Health Sciences, Addis Ababa, Ethiopia,; 2 Department of Pathology, University of Gondar College of Medicine and Health Sciences, Gondar, Ethiopia

**Keywords:** Histopathological pattern, bone tumors, location, age, sex

## Abstract

**Introduction:**

there is few data on epidemiology or clinico-pathology of primary bone tumours in Ethiopia. Bone tumors often have a similar presentation and clinical approach, but they present individual characteristics that are important for treatment and prognosis. This study aimed to provide a brief overview of histopathological pattern of primary bone tumours and the distribution of specific diagnosis by age, sex and anatomic locations.

**Methods:**

a retrospective descriptive cross-sectional study was conducted from January 2015 to October 2019 in the Department of Pathology, College of Health Sciences, Addis Ababa University based on surgical biopsy results.

**Results:**

there were 335 patients; 158 (47%) had benign tumours and 177 (53%) had malignant tumours. Osteochondroma was the most common benign bone tumour with 32.3% followed by giant cell tumour 16.5%, chondroma 11% and others 40.2%. Osteosarcoma constitutes 62% of all primary malignant bone neoplasms, followed by ewing sarcoma 15.2%, chondrosarcoma 11.3%, and others 11.5%. Most age group affected by primary bone tumours were 10-29 yrs and most common locations of presentation for primary malignant bone tumours were distal femur and proximal tibia.

**Conclusion:**

the present study results parallel the findings previously reported in literature and show a similar distribution of primary bone tumors as in the other developed and underdeveloped countries.

## Introduction

Tumors of the skeletal system are relatively constant in their pattern of presentation [1]. The five basic parameters of importance in this regard are the age of the patient, bone involved, specific area within the bone (epiphysis, metaphysis, or diaphysis; cortex, medulla, or periosteum), radiographic appearance, and microscopic appearance. The pathologist should be fully aware of the first four before trying to evaluate the fifth [2]. Otherwise, serious mistakes may occur.

In a series of pattern of bone tumour seen at Addis Ababa University, Ethiopia, there were 51.3% males and 48.7% females with histopathological tissue diagnosis of the bone tumor. The ages ranged from 7 to 55 years with a peak in the 15-29 years age group. The commonest primary malignant tumour was osteosarcoma 28.5%. Exostosis was second [3]. In a review of histopathological pattern of primary bone tumours and tumour-like lesions in Ile-Ife, Nigeria; osteochondroma was the most common benign tumour seen and accounted for 32.1% of all benign bone tumours. The tibia was the commonest site, contributing 33.3% of all cases. Osteosarcoma was the most common primary malignant bone tumour seen, 42%. The mean age for osteosarcomas was 21.33 years. Also, 42.86% occurred in the distal third of the femur, 14.28% in the distal tibia, 14.28% in the proximal tibia, 9.52% in the mandible and in the proximal third of the femur, the maxilla, humerus and calcaneus 4.76% each [4].

The incidence rates of specific bone sarcomas are age-related and as a group, have a bimodal distribution. The first well-defined peak occurs during the second decade of life, while the second occurs in people aged > 60 years. Up to 43% of malignant bone tumours will arise around the knee, but under the age of 20 years this rises to 56%. The second most common site is the pelvis, which is numerically the most common site of presentation for both ewing sarcoma and chondrosarcoma [5]. This study aimed to provide a brief overview of histopathological pattern of primary bone tumours and the distribution of specific diagnosis by age, sex and anatomic locations.

## Methods

**Study design and setting:** this is a retrospective descriptive cross-sectional study which was based on surgical biopsy specimens submitted to the Pathology Department, Faculty of Medicine, Addis Ababa University for histopathologic examination.

**Study population:** all bone tumor patients, who presented to the Department of Pathology at Black Lion Hospital (BLH) between the study periods of January 1^st^ 2015 and October 31^st^ 2019 were recruited. Three hundred thirty five histologically proven primary tumors of bone were included in this study. Inadequate data entries, duplicate registrations, diagnoses such as small round blue cell tumors, high grade sarcoma were removed from the study. Ten patients with secondary carcinoma of bone were also excluded. Maxillofacial bone tumours were also included in this study.

**Data collection:** all surgical biopsy results in the files of the Department of Pathology were reviewed by the author in order to identify all patients with histologically proven primary bone tumor. The clinical data and radiologic diagnosis were utilized for accurate interpretation of biopsy material. Haematoxylin and Eosin were used as the routine stains of the microscopic section in all cases. All tumours were analyzed according to age, sex, skeletal localizations and histologic types. For histology typing of bone tumours, the World Health Organization (WHO) classification of tumours of bone was used.

**Definitions:** primary bone tumors were dependent variable while age, sex and anatomic locations were independent variables in this study.

**Statistical analysis:** the data were entered and analysed, based on the histological types of tumours and their distribution according to age, sex and skeletal location using statistical software, SPSS version 23. The mean age of presentation for the most common primary bone tumours were calculated. This is purely a descriptive study and no statistical association test performed (analytical component was not done).

**Ethical considerations:** ethical clearance was obtained from research and ethics committee of the Department of Pathology, College of Health Sciences, Addis Ababa University with a reference number of MF/Patho/170/2019.

## Results

**General characteristics:** of the 335 patients who had primary tumors of bone, 158 (47%) were benign and 177 (53%) were malignant. One hundred and fifty-three (46%) were females and 182 (54%) were males in this study. Primary bone tumours involved all age groups but the majority of primary bone tumours occurred in patients between 10 and 29 years of age.

**Clinical presentations:** the most common presenting symptom was an enlarging soft tissue mass. Other common presenting symptoms included local pain and pathologic fracture. Occasionally, systemic signs such as weight loss, fatigue, anorexia, fever, dyspnea, or night sweating were also found. Duration of symptoms before presentation for benign and malignant bone tumors ranged from 1 month to 10 years and 1/2 months to 3 years respectively.

**Histopathological features:** osteochondroma was the most common benign bone tumour with 32.3% followed by giant cell tumour 16.5%, chondroma 11%, fibrous dysplasia 10%, osteoma 9%, and others 21.2%. Osteosarcoma 109 (62%) were the most common malignant tumours of bone followed by ewing sarcoma 27 (15.2%) followed by chondrosarcoma 20 (11.3%), 17 (9.5%) multiple myeloma and plasmacytoma, 4 (2%) angiosarcoma and chordoma.

**Association of age and sex with histopathological features:** as shown in Table 1, 110 (69.6%) benign bone tumours occurred in patients between 10 and 29 years of age. There were 72 (46%) male and 86 (54%) female patients with benign tumours of bone. As shown in Table 2, 118 (66.6%) malignant bone tumours occurred in patients between 10 and 29 years of age. There were 110 (62%) male and 67 (38%) female patients with malignant tumours of bone. The distribution of primary bone tumours by histologic type and sex of patient is listed in Table 3 and Table 4.

**Table 1:** distribution of benign tumours of bone by histologic type and age of patient as seen in the Department of Pathology, Faculty of Medicine, Addis Ababa University, 2015-2019

Histologic type	Age group in yrs								Total no. of cases
	0-9	10-19	20-29	30-39	40-49	50-59	60-69	70-79	
Osteochondroma	3	35	9	1	2	1	0	0	51
Chondroma	1	10	2	4	1	0	0	0	18
Chondromyxoid fibroma	0	1	0	0	0	0	0	0	1
chondroblastoma	0	1	0	0	0	0	0	0	1
BPOP	0	0	0	2	0	0	0	0	2
Synovial chondromatosis	0	0	0	1	1	0	0	0	2
Ossifying fibroma	0	3	6	0	0	0	0	0	9
Fibrous dysplasia	3	2	8	1	2	0	0	0	16
Osteofibrous dysplasia	1	0	0	1	0	0	0	0	2
Non-ossifying fibroma	4	1	0	0	0	0	0	0	5
GCT	1	4	10	2	1	1	0	0	19
GCT with ABC	0	3	3	0	1	0	0	0	7
ABC	1	5	0	1	1	0	0	0	8
Osteoma	0	2	4	4	3	1	0	0	14
Osteoblastoma	0	1	0	0	0	0	0	1	2
Hemangioma	0	0	0	0	0	1	0	0	1
Total	14	68	42	17	12	4	0	1	158

BPOP: bizarre parosteal osteochondromatous proliferation; GCT: giant cell tumour; ABC: aneurysmal bone cyst

**Table 2:** distribution of malignant tumours of bone by histologic type and age of patient as seen in the Department of Pathology, Faculty of Medicine, Addis Ababa University, 2015-2019

Histologic type	Age group in years								Total no. of cases
	0-9	10-19	20-29	30-39	40-49	50-59	60-69	70-79	
Osteosarcoma	4	58	34	7	4	1	1	0	109
Chondrosarcoma	0	0	3	3	2	6	4	2	20
Multiple myeloma	0	0	0	0	1	1	1	1	4
Plasmacytoma	0	0	0	1	5	5	1	1	13
Ewing sarcoma	3	16	7	1	0	0	0	0	27
Angiosarcoma	0	0	0	0	1	0	1	0	2
Chordoma	0	0	0	0	0	2	0	0	2
Total	7	74	44	12	13	15	8	4	177

**Table 3:** distribution of benign tumours of bone by histologic type and sex as seen in the Department of Pathology, Faculty of Medicine, Addis Ababa University, 2015-2019

Benign bone tumors	No. of males	Percent (%)	No. of females	Percent (%)	Total no. of cases	Percent (%)
Osteochondroma	27	53%	24	47%	51	32.3%
Chondroma	6	33%	12	67%	18	11%
Chondromyxoid fibroma	1	100%	0	0%	1	0.6%
Chondroblastoma	1	100%	0	0%	1	0.6%
Synovial chondromatosis	0	0%	2	100%	2	1.3%
BPOP	2	100%	0	0%	2	1.3%
Osteoma	5	36%	9	64%	14	9%
Osteoblastoma	1	50%	1	50%	2	1.3%
Ossifying fibroma	1	11%	8	89%	9	6%
Fibrous dysplasia	6	37.5%	10	62.5%	16	10%
Osteofibrous dysplasia	1	50%	1	50%	2	1.3%
Non-ossifying fibroma	3	60%	2	40%	5	3.2%
Giant cell tumor	12	63%	7	37%	19	12%
GCT with ABC	2	29%	5	71%	7	4.5%
ABC	4	50%	4	50%	8	5%
Hemangioma	0	0%	1	100%	1	0.6%
Total	72	46%	86	54%	158	100%

BPOP: bizarre parosteal osteochondromatous proliferation; GCT: giant cell tumour; ABC: aneurysmal bone cyst

**Table 4:** distribution of primary malignant tumours of bone by histologic type and sex as seen in the Department of Pathology, Faculty of Medicine, Addis Ababa University, 2015-2019

Malignant bone tumors	Number of males	Percent (%)	Number of females	Percent (%)	Total number of cases	Percent (%)
Osteosarcoma	65	60%	44	40%	109	62%
Chondrosarcoma	11	55%	9	45%	20	11.3%
Plasmacytoma	8	62%	5	38%	13	7.3%
Plasma cell myeloma	4	100%	0	0%	4	2.2%
Ewing sarcoma	19	70%	8	30%	27	15.2%
Angiosarcoma	1	50%	1	50%	2	1%
Chordoma	2	100%	0	0%	2	1%
Total	110	62%	67	38%	177	100%

**Association of skeletal location with histopathological features:** the skeletal distribution of benign and malignant primary tumours of bone is listed in Table 5. Of the 158 benign tumours of bone, 58 (37%) involved distal femur and proximal tibia; 32 (20%) involved short tubular bones of the hands and feet; 22 (14%) involved craniofacial bones. Osteochondroma occurred mainly in distal femur and proximal tibia; while chondroma involved mainly short tubular bones of the hands and feet. Of the 177 malignant tumours of bone 92 (52%) involved distal femur, proximal tibia and fibula; 28 (16%) pelvic bones mainly ilium, 19 (11%) shoulder girdle, 18 (10%) trunk, 20 (11%) involved jaw bones, skull bones, lower leg and upper limb bones. The metaphysical part of the long bones was the predilection site for osteosarcomas; 72% in this series occurred in distal femur and proximal tibia.

**Table 5:** skeletal distribution of benign and malignant tumours of bone as seen in the Department of Pathology, Faculty of Medicine, Addis Ababa University, 2015-2019

	Skeletal location in percentage (%)								
Benign bone tumors	Knee*	Pelvis#	Shoulder girdle	Lower leg	Upper limb	Trunk	Face and skull bone	Jaw bone	Others
Osteochondroma	60%	8%	10%	2%	6%	4%	0%	0%	10%
Chondroma	11%	0%	0%	0%	0%	0%	0%	0%	89%
Chondromyxoid fibroma	100%	0%	0%	0%	0%	0%	0%	0%	0%
Chondroblastoma	0%	0%	100%	0%	0%	0%	0%	0%	0%
Synovial chondromatosis	100%	0%	0%	0%	0%	0%	0%	0%	0%
BPOP	0%	0%	0%	0%	0%	0%	0%	0%	100%
Ossifying fibroma	0%	0%	0%	0%	0%	0%	44%	56%	0%
Fibrous dysplasia	13%	0%	6%	0%	0%	6%	19%	56%	0%
Osteofibrous dysplasia	0%	0%	0%	100%	0%	0%	0%	0%	0%
Non-ossifying fibroma	100%	0%	0%	0%	0%	0%	0%	0%	0%
Giant cell tumor	37%	5%	0%	11%	5%	0%	0%	5%	37%
GCT with ABC	86%	14%	0%	0%	0%	0%	0%	0%	0%
Aneurysmal bone cyst	12.5%	25%	25%	0%	0%	25%	0%	0%	12.5%
Osteoma	0%	0%	0%	0%	0%	0%	100%	0%	0%
Osteoblastoma	100%	0%	0%	0%	0%	0%	0%	0%	0%
Hemangioma	0%	0%	0%	0%	0%	0%	100%	0%	0%
All diagnoses	37%	5%	6%	3%	3%	3%	14%	9%	20%
**Malignant bone tumors**									
Osteosarcoma	72%	9%	8%	0%	4%	2%	0%	5%	0%
Chondrosarcoma	20%	30%	25%	5%	5%	15%	0%	0%	0%
Multiple myeloma	0%	25%	0%	0%	0%	25%	25%	25%	0%
Plasmacytoma	23%	0%	8%	8%	0%	53%	0%	8%	0%
Ewing sarcoma	22%	33%	15%	7%	4%	19%	0%	0%	0%
Angiosarcoma	0%	50%	0%	0%	50%	0%	0%	0%	0%
Chordoma	0%	100%	0%	0%	0%	0%	0%	0%	0%
All diagnoses	52%	16%	11%	2%	4%	10%	1%	4%	0%

Data from 335 primary bone tumours seen at Black Lion Specialized and Teaching Hospital, Ethiopia; *knee tumors include distal femur, proximal tibia and proximal fibula; #pelvis tumors include pelvis and proximal femur locations; shoulder girdle tumors include proximal humerus, clavicle and scapula; trunk includes vertebrae, ribs, sternum etc.; others include ankle joint, short tubular bones of the hands and feet

## Discussion

According to estimates of cancer incidence in Ethiopia in 2015 using population-based registry data, the crude incidence rate of bone and cartilage tumors per 100,000 population among men and women was 0.6 and 1.0 respectively [6]. In the present series of histopathological pattern of primary tumours of bone and the distribution of specific diagnosis by age, sex and anatomic locations; there were 335 patients who had primary tumors of bone, 158 (47%) were benign and 177 (53%) were malignant. Osteochondroma was the most common benign bone tumour and osteosarcoma were the most common malignant tumours of bone. Most age group affected by primary bone tumours were 10-29 yrs and most common locations of presentation for primary malignant bone tumours were distal femur and proximal tibia.

In the present study malignant bone tumours outnumbered benign bones tumours. This may be due to benign tumours of bone and articular cartilage such as osteochondromas and chondromas are usually asymptomatic and many of those that are identified are never diagnosed, and/or failure of surgeons to submit all excised bone tissues to pathology department so that the true incidence of benign bone tumors is much greater than seen in this study. Osteochondromas are by far the most common benign lesions observed accounting for 32.3% of all benign bone tumours. The youngest was five-year-old boy and the oldest a 52-year-old man, with an average age of 19.17 years. Giant cell tumors were the second most common benign tumours encountered in this study accounting for 16.5% of all benign bone tumours. The average patient age was 24.23 years. In a review of 117 cases of bone tumors in a tertiary care hospital of South India, osteochondroma was the most common accounting for 22.22% followed by giant cell tumor 20.51% [7]. Chondromas were the third most prevalent benign tumours encountered in this study accounting for 11% of all benign bone tumours.

Fibrous dysplasia and ossifying fibroma accounted for 10% and 6% of all benign bone tumours respectively. Seventy-five percent of fibrous dysplasia arose in craniofacial bones. Fifty-six percent of ossifying fibroma arose in jaw bones, while 46% in craniofacial bones. Osteomas accounted for 9% of all benign bone tumours. One hundred percent of the lesions arose in craniofacial bone. In a series of bone and articular cartilage tumours as seen in the Department of Pathology, Faculty of Medicine, Addis Ababa University, there were 689 patients; 400 (58%) had benign tumours and 289 (42%) had malignant tumours. Osteochondroma was the most common benign bone tumour with 36.5% followed by chondroma at 20.5%. Osteosarcoma was the most common subtype encountered, accounting for 35.1% of all primary malignant bone neoplasms followed by chondrosarcoma 27.1%, ewing´s sarcoma 11.1%. The age, sex, and skeletal distribution followed the same pattern as in the present study [8].

Osteosarcoma was the most common subtype encountered in this study, accounting for 62% of all primary malignant bone neoplasms. The youngest was six-year-old boy and the oldest a 60-year-old woman, an average age of 20.92 years. Seventy two percent of the lesions arose in distal femur and proximal tibia. Out of 1,238 subjects diagnosed with primary bone cancers in Tiwan between 2003 and 2010, osteosarcoma was the most common subtype, accounting for 45% followed by chondrosarcoma 18%, and ewing sarcoma 8% [9]. Ewing sarcomas were the second most common malignant tumours encountered in this study accounting for 15.2% of all malignant bone tumours. The youngest was 7-year-old boy and the oldest a 30-year-old man, with an average age of 16 years.

Fifty-five percent of the lesions arose in pelvic bone mainly ilium, distal femur and proximal tibia. Out of 146 subjects diagnosed with primary bone cancers in Romania between 2005 and 2013, osteosarcoma was the most common subtype, accounting for 54.1% followed by Ewing´s sarcoma, 30.82% and chondrosarcoma, 8.9%. The average patient age was 13.32 years. The most common anatomical distribution of the tumors was femur, 32.19%, tibia, 25.34% and humerus, 11.64% [10].

Chondrosarcomas were the third most common malignant tumours encountered in this study accounting for 11.3% of all malignant bone tumours. The average patient age was 50.35 years. About 75% of chondrosarcomas arose in the long bones of the lower and upper extremities and pelvic bones mainly ilium. In a series of characteristics of benign and malignant bone tumors registered in the Hiroshima tumor tissue registry, 1973-2012, the most frequent malignant bone-tumor types were osteogenic tumors 39.7% and chondrogenic tumors 26.7%. About 41.7% occurred in the long bones of the lower limb [11].

Solitary plasmacytoma of bone and plasma cell myeloma accounted for 7.3% and 2.2% of all malignant bone tumours respectively in this study. The other rare malignant primary bone tumors-encountered in this series were chordoma arose on sacrococcygeal bone and angiosarcoma arose on proximal humerus and femur. This study further contributes to what is already known regarding the histopathological patterns of primary bone tumours and creates a paradigm for future studies of bone tumours in our environment. Limitations encountered include sociodemographic factors were not fully documented on patients´ files of department of pathology as well as our inability to carry out further high-end histopathological analysis due to dearth of requisite technology.

## Conclusion

Osteochondroma was the most common benign bone tumour and osteosarcoma was the most common malignant bone tumour. Most age group affected by primary bone tumours were 10-29 yrs and most common locations of presentation for primary malignant bone tumours were distal femur and proximal tibia. The present study results parallel the findings previously reported in literature and show a similar distribution of primary bone tumors as in the other developed and underdeveloped countries.

### What is known about this topic

- *There is few data on epidemiology or clinico-pathology of primary bone tumours in Ethiopia;*
- *Common locations of presentation for primary malignant bone tumours were distal femur and proximal tibia*.

### What this study adds

- *Figures are now available and will serve as a template upon which future studies in this field can be built;*
- *This study also provides the histopathological pattern of bone tumours which is not widely studied*.
